# LncRNAs in domesticated animals: from dog to livestock species

**DOI:** 10.1007/s00335-021-09928-7

**Published:** 2021-11-13

**Authors:** Sandrine Lagarrigue, Matthias Lorthiois, Fabien Degalez, David Gilot, Thomas Derrien

**Affiliations:** 1 INRAE, INSTITUT AGRO, PEGASE UMR 1348, 35590 Saint-Gilles, France; 2 Univ Rennes, CNRS, IGDR (Institut de Génétique et Développement de Rennes) - UMR 6290, 2 av Prof Leon Bernard, F-35000 Rennes, France; 3 CLCC Eugène Marquis, INSERM, Université Rennes, UMR_S 1242, 35000 Rennes, France

## Abstract

Animal genomes are pervasively transcribed into multiple RNA molecules, of which many will not be translated into proteins. One major component of this transcribed non-coding genome is the long non-coding RNAs (lncRNAs), which are defined as transcripts longer than 200 nucleotides with low coding-potential capabilities. Domestic animals constitute a unique resource for studying the genetic and epigenetic basis of phenotypic variations involving protein-coding and non-coding RNAs, such as lncRNAs. This review presents the current knowledge regarding transcriptome-based catalogues of lncRNAs in major domesticated animals (pets and livestock species), covering a broad phylogenetic scale (from dogs to chicken), and in comparison with human and mouse lncRNA catalogues. Furthermore, we describe different methods to extract known or discover novel lncRNAs and explore comparative genomics approaches to strengthen the annotation of lncRNAs. We then detail different strategies contributing to a better understanding of lncRNA functions, from genetic studies such as GWAS to molecular biology experiments and give some case examples in domestic animals. Finally, we discuss the limitations of current lncRNA annotations and suggest research directions to improve them and their functional characterisation.

## Introduction

The last decade has witnessed the importance of the non-coding genome in the exhaustive characterization of genotype to phenotype relationships. Beside traditional protein-coding genes (mRNAs), animal genomes are pervasively transcribed into a myriad of short and long non-coding RNAs (Carninci 2005; Djebali et al. 2012; Mattick and Rinn 2015; Snyder et al. 2020) with various regulatory functions. Among these, long non-coding RNAs (lncRNAs) represent a vast and heterogeneous class of genetic elements with specific features in comparison with mRNAs. By definition, lncRNAs display very low coding-potential capabilities and are more tissue-specific and nuclear enriched than protein-coding genes (Cabili et al. 2011; Derrien et al. 2012). However, similar to mRNAs, they exert a variety of functions at either the transcriptional or posttranscriptional levels in cis or in trans (Ponting et al. 2009; Gil and Ulitsky 2019; Statello et al. 2021).

Given the interest for mapping to genomic regions the morphological, agronomical, or behavioural traits of domesticated animals, researchers have traditionally used genome-wide association studies (GWAS) to identify common polymorphisms associated with phenotypes of interest (Buniello et al. 2019). Yet, as in humans, many of the trait-associated variations identified by GWAS fall within non-coding intervals of the genome, reinforcing the need to deeply characterise the regulatory regions of domesticated species. Concomitantly, advances in high-throughput transcriptome sequencing technologies (RNAseq) has enabled the systematic exploration of this uncharacterised genomic space, first in human and model organisms (Djebali et al. 2012; Breschi et al. 2017) and more recently in other canonical and non-canonical organisms (Brown et al. 2014; Tagu et al. 2014). By combining RNAseq in numerous tissues or cell lines and at different developmental stages, it is now feasible to develop near comprehensive maps of coding and non-coding transcribed regions in order to refine the interpretation of genotype to phenotype studies in homogeneous populations of domesticated animals.

Here, we review the current knowledge about lncRNAs mainly in dog, horse, cow, pig, and chicken chosen as main domesticated species and compare these lncRNA maps with respect to best-studied species in research such as human and mouse. The domestic dog (*Canis lupus familiaris*) is an exceptional case of species for tracking down genotype to phenotype relationships because pet dogs exhibit the most extreme phenotypic variations observed in terrestrial animals (Ostrander et al. 2017). This has been attributed to the particular history of dogs, from initial domestication events (> 14kya) of a now extinct grey wolf (*Canis lupus*) (Frantz et al. 2016) followed by intense breeding practices that led to the creation of modern purebred breeds during the Victorian era. However, this artificial selection for esthetical or behavioural traits has also led to the co-selection of morbid alleles that are now making dog breeds particularly predisposed to Mendelian diseases and cancers (Steenbeek et al. 2016). Dogs therefore represent an ideal genetic system to study phenotypically plastic traits and disease/cancer-related loci (Karlsson and Lindblad-Toh 2008). The pig, chicken and cow are livestock species which are the most used sources of animal protein worldwide, for the meat with 121, 114 and 67 Million tonnes produced worldwide and other products, e.g. eggs with 80 Million tonnes produced worldwide by laying hens (Food and Agriculture Organization of the United Nations 2021). These three species have been selected for multiple traits related to production (in terms of quantity and quality), efficiency, productive longevity, fertility, resilience, animal welfare and health. Among these three species, chicken has a particular status because of its phylogenetic characteristics, as birds and mammals diverged 300 mya. Finally, the horse (*Equus caballus*) is a key domesticated animal (~ 5 kya ago) from both cultural and economic aspects (Kalbfleisch et al. 2018) and has been selected for multiple traits (endurance, speed, appearance…).

For all these domesticated species, growing catalogues of long non-coding RNAs are being characterised, leading to increased examples of the association of lncRNAs with phenotypic traits of interest. However, lncRNA loci are still incomplete compared with protein-coding gene catalogues, partly due to the biological properties of lncRNAs. Therefore, only a handful of lncRNAs in domesticated animals have been associated with a probable causative effect or have been functionally validated. We thus emphasise the need to integrate complementary approaches for better annotating lncRNAs and for functionally validating trait-associated non-coding elements in the study of genotype to phenotype relationships.

## Annotation of long non-coding RNAs in domesticated species

Transcriptome sequencing has revolutionized the process of genome annotation (Zhong Wang et al. 2009). RNAseq can be used to target different RNA populations of the cells, either with or without polyA tails. Except for a few studies mostly in human cells (Djebali et al. 2012), most of the annotated lncRNAs so far in pets and livestock species have been extracted from protocols employing polyA RNA selection. Once transcriptome sequences are available and quality-controlled, the bioinformatic process of annotating long non-coding RNAs basically involves three major steps (Table 1). The first one consists in *mapping* transcriptomic data (ESTs, cDNAs and now short and long RNAseq reads) onto a reference genome using a splice-aware mapper (e.g. STAR (Dobin et al. 2013)) in order to correctly model exon–intron junctions (Djebali et al. 2017). The second step aims at assemble mapped reads into known (already present in the reference annotation) and novel transcripts using dedicated *transcript reconstruction* tools [*e.g.* Cufflinks (Trapnell et al. 2010) or StringTie (Pertea et al. 2015)]. While the two first steps are common to both coding and non-coding genes, the third step focuses on classifying novel transcripts into mRNAs or lncRNAs by computing their *coding-potential* capabilities. An additional though optional step would involve the sub-classification of newly annotated lncRNAs with respect to the localisation and the direction of transcription of proximal mRNA transcripts in order to define lncRNAs classes such as lincRNAs (long intergenic ncRNAs) or antisense lncRNAs.

**Table 1:** Bioinformatic tools for annotating and classifying lncRNAs from multi-species databases

Database name	Read mapping	Gene model-ling	Coding-potential assesse-ment	Number of lncRNA genes/transcripts by species (*genome assembly version)*						
				Human	Mouse	Cow	Pig	Chicken	Dog	Horse
Ensembl (v104)	BWA	Exone-rate	ORF and PFAM align-ment^a^	16 896/46 960 (GRC g38.p13)	9972/12 601 (GRC m39)	1488/2199 (ARS_ UCD1.2)	6979/9367 (Sscrofa 11.1)	5506/8870 (GRC g6a)	7083/12 283 (Can Fam 3.1)	7244/11 978 (Equ Cab 3.0)
NCBI (v105)	Minimap2 (long read) Spilign (short-read)	Gnomon	Gnomon	16 375/27 838 (GRC g38.p13)	13 317/23 542 (GRC m39)	5183/7254 (ARS_ UCD1.2)	5605/9292 (Sscrofa 11.1)	5147/8233 (GRC g6a)	10 823/19 248 (Can Fam 3.1)	6789/10 850 (Equ Cab 3.0)
NONCODE (v6.0)	Literature parsing with RNAseq key words + "CuffCompare" to deal with overlapping features		Comparison with RefSeq + "CNIT"	96 411/173 112 (GRC g38)	87 890/131 974 (GRC m39)	22 127/23 515 (UMD 3.1)	17 811/29 858 (Sscrofa 10.2)	9527/12 850 (galgal4)	NA	NA

The number of lncRNAs found for each species with the corresponding assembly used is also presented

^a^Gene models containing a substantial open reading frame (ORF) and protein domains (e.g. from Pfam) are classified as coding. For human and mouse annotations, additional manual curations from Gencode

### Based on dedicated annotation resources

LncRNA maps of domesticated species can be reached from several publicly available resources. As shown in Table 1, these resources use different computational tools at each main step of the RNAseq processing pipeline described above (Table 1). Furthermore, the total number of lncRNA genes and transcripts vary substantially between domesticated species and do not currently scale with the number of lncRNA in human and mouse catalogues (Table 1).

One of the most widely used resources for extracting gene annotations is provided by the Ensembl genome browser (Aken et al. 2016; Howe et al. 2021). Ensembl provides genome-wide annotations of protein-coding and non-coding RNAs for more than 250 vertebrates, including many domesticated animals. In human or canonical model organisms (*e.g.* mouse), the specific process of annotating long non-coding RNAs combines automated annotation from RNAseq data processed by the Ensembl gene build pipeline and manual curation by the HAVANA/Gencode group (Frankish et al. 2019). The Gencode database (version 37), which is synchronised with Ensembl, has compiled 17 948 human lncRNA genes (~ 48 000 transcripts) and 13,186 mouse lncRNA genes (~ 18 000 transcripts) (version M26). For other species, including domesticated animals, the description of the built lncRNA catalogues has been less detailed to date and does not include manual curation which most likely impacts the quality of these annotations. In addition, in contrast to human and mouse Ensembl catalogues, only intergenic genes (lincRNAs) are referenced, meaning that other biotypes such as antisense exonic or sense intronic transcripts, are not reported for domesticated species.

**Fig. 1:**
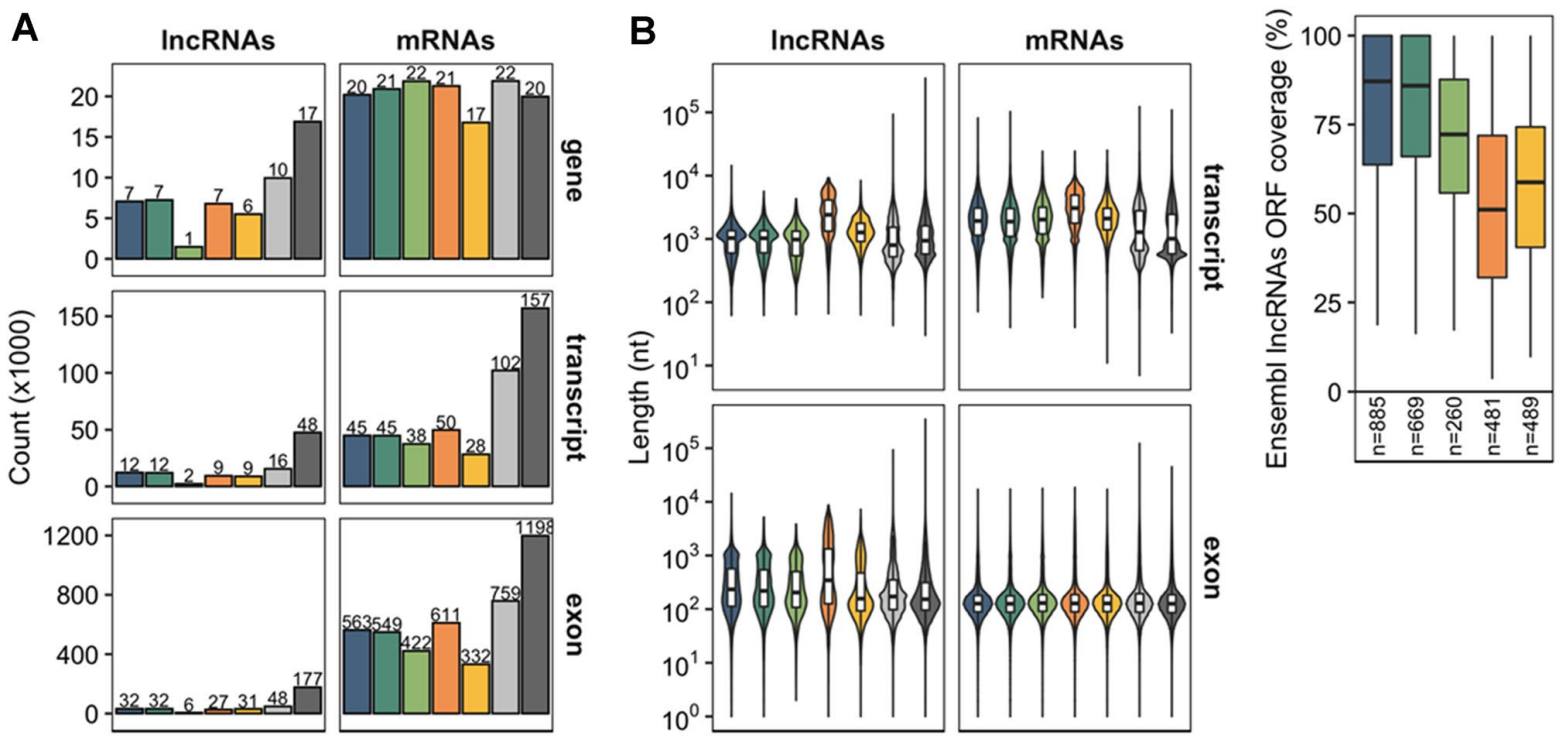
Characterization of lncRNA and mRNA gene structures in 5 domesticated animals (dog, horse, cow, pig, and chicken, respectively in dark green, orange, purple, pink, and light green) in comparison with mouse and human annotations (light and dark grey respectively) extracted from Ensembl (v103). **A** Comparison of the number of lncRNA and mRNA genes, transcripts, and exons (number of lncRNA and mRNA features are indicated on top of each bar). **B** Boxplot distributions of the length of lncRNA and mRNA transcripts and exons. **C** ORF coverage of Ensembl-based lncRNAs annotated as protein-coding by the FEELnc program

The number of Ensembl lncRNA genes varies greatly between the 5 major domesticated species. For instance, 1480 lncRNA genes have been identified in the cow and approximately 7000 in the horse, dog, and pig, whereas the number of protein-coding genes (mRNAs) remains more stable (~ 20,000) (Fig. 1A). Similar to mouse, the number of lncRNA transcripts/isoforms per gene in the cow and dog ranges from 1.4 to 1.7 lncRNA transcripts per gene, respectively, which is significantly lower than the 1.8 and 2.5 mRNA transcripts per gene for protein-coding genes in the respective species. This might be due to the difficulty to identify lowly expressed lncRNA isoforms by RNAseq methodologies (Fig. 1A). When comparing the length of lncRNA transcript sequences across domesticated species (Fig. 1B), one could note that pig and chicken lncRNA transcripts are significantly longer than those in other mammal species (Mann–Whitney *U* tests, *p* values < 2.2e-16). Interestingly, the recent annotations of the new *sus scrofa* and *gallus* assemblies have benefited from the use of long-read RNAseq (LR-RNAseq) (PacBio Iso-Seq from nine adult porcine tissues (Warr et al. 2020; Beiki et al. 2019) and from originally two and now six addition chicken tissues (Kuo et al. 2017; https://www.ensembl.org/Gallus_gallus/Info/Annotation), which might have enabled global extensions of transcript models as this trend has also been observed for protein-coding genes (Fig. 1B).

As every automatic modelling process, the Ensembl gene build pipeline might also suffer from incorrect annotations. A closer inspection of the Ensembl-based catalogues of lncRNAs in the five domesticated species identified the probable misclassification of some mRNAs as long non-coding transcripts. For instance, between 5.5% lncRNAs in horse and 11.8% lncRNAs in cow were classified as protein-coding by the FEELnc program (Wucher et al. 2017). When searching for the longest ORFs, either partial (*i.e.* missing start codon) or full (having both a start and stop codons), in these "ambiguous" transcripts (Fig. 1C), the ORF appears to cover a large fraction of the annotated RNA sequences (median = 51% in pig to 82% in dog) despite the fact that it should have been filtered (Aken et al. 2016) (Table 1). Therefore, a high ORF coverage would suggest that these transcripts might represent bona fide protein-coding transcripts and exclude the possibility that they correspond to lncRNAs harbouring small ORFs (smORFs) (Bazzini et al. 2014; Ruiz-Orera et al. 2014).

Despite these shortcomings, the Ensembl resource is extremely useful for the scientific community working on non-model organisms because it provides a versioned, stringent, and freely available set of gene/transcript structures (both coding and non-coding) at the basis of most downstream bioinformatic analyses.

Besides Ensembl, several more recent databases also provide extensive annotations of non-coding genes based on different computation pipelines (Table 1). For instance, the NONCODE database (Zhao et al. 2016) is specifically dedicated to the annotation and bioinformatic characterization of long non-coding RNAs in animals and plants. The integration of lncRNAs in NONCODE makes use of the CuffCompare tool from Cufflinks (Trapnell et al. 2010) in order to combine and filter multiple sources of lncRNA annotations. One advantage of NONCODE over Ensembl is that it involves the use of a published coding-potential assessment tool, CNIT for Coding-Non-Coding Identifying Tool (Guo et al. 2019), an updated version of the CNCI program (Sun et al. 2013), to discriminate reconstructed coding from non-coding gene models. One limitation though is that NONCODE only includes lncRNA catalogues for 16 animal species, excluding dog and horse for instance. Whereas, in the case of Ensembl-matched species, the number of lncRNA transcripts is significantly higher with 9527, 17,811, and 22,227 lncRNA loci for chicken, pig, and cow, respectively. In addition, NONCODE provides a detailed characterization of annotated lncRNAs based on phylogenetic conservation, disease association, as well as lncRNAs overlapping SNPs/GWAS hits. Historically, the first specific database of lncRNAs dedicated to livestock species was the domestic-animal lncRNAs database (ALDB) (Li et al. 2015), although this database seems not to have been updated since 2016. Using a rather out-dated bioinformatic pipeline including the TopHat mapper and the CPC tool for assessing coding-potential, ALDB comprises 6151 (8923), 7381 (12 103), and 5213 (8250) lincRNA loci (transcripts) for chicken, pig, and cow, respectively. Finally, it is also worth mentioning the NCBI reference sequence database (RefSeq) that provides automatic annotation of lncRNAs and mRNAs in > 55,000 organisms, including domesticated species. In particular, NCBI/RefSeq makes use of the "eukaryotic genome annotation pipeline" with the Gnomon program, which combines homology searching with ab initio modelling (O’Leary et al. 2016) and comprises 10,823, 5 147, 5 605, and 5 183 lncRNA loci in dog, chicken, pig, and cow, respectively (Table 1).

**Fig. 2:**
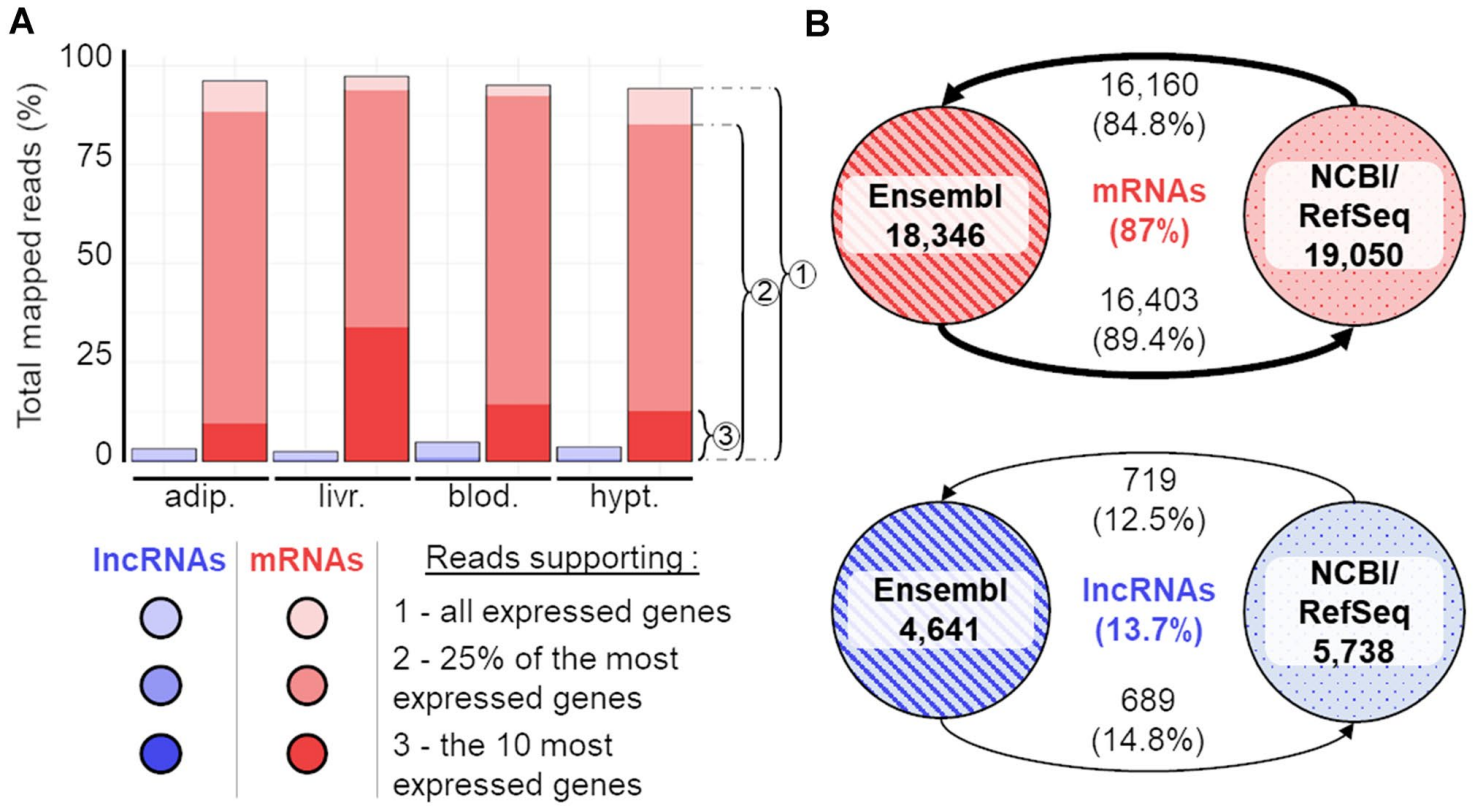
Distribution of reads supporting lncRNAs and mRNAs (**A**) and gene overlap between NCBI and Ensembl resources according to both biotypes (**B**). **A** For each gene biotype (lncRNAs in blue and mRNAs in red), the dark, intermediate and light shades correspond to the percentage of reads supporting all expressed genes, 25% of the most expressed genes and the 10 most expressed genes respectively. RNAseq data correspond to the chicken PRJEB28745 project and 4 tissues (*adip* adipose tissue, *livr* liver, *blod* blood, *hypt* hypothalamus) of the same population (Rhode Island Red). B) Percentages of chicken lncRNA gene overlap—using 1 bp or more—between the GRCg6a—V104 Ensembl and NCBI gene catalogues. Note that these overlaps have been computed at the gene level given the uncertainty of isoform modelling with short-reads as explained in the main text

Although these publicly available catalogues represent a rich resource for digging into trait-associated loci, involving annotated lncRNAs, a limited genomic overlap still exist between these annotations (Fig. 2), most likely reflecting the high specificity of lncRNA expression profiles and the different origins of the input transcriptomic sequencing data.

## De novo transcriptome reconstruction of new long non-coding RNAs

The democratization of RNAseq combined with efficient bioinformatic tools to rapidly process transcriptome data have allowed researchers working on domesticated species to build their own catalogues of lncRNAs.

### Long non-coding RNA studies and atlas in dogs

The scientific community provided a first dog reference genome assembly, together with an annotation of ~ 20,000 protein-coding genes, of a boxer breed in 2005, making the dog the fifth mammal to be sequenced (Lindblad-Toh et al. 2005). However, a comprehensive catalogue of coding and non-coding/regulatory elements for the interpretation of the many GWAS signals lying outside of annotated mRNAs and for the eventual identification of the actual causal mutations was not provided until 2014. At that time, Hoeppner and colleagues combined RNAseq data from 10 distinct canine tissues to build ~ 7200 lincRNA transcripts and 4600 antisense lncRNAs (Hoeppner et al. 2014). In 2017, thanks to the collection of novel canine RNA samples provided within the framework of the European LUPA consortium (Lequarré et al. 2011), Wucher et al. integrated 20 additional RNAseq data to build a new canine reference annotation (Wucher et al. 2017). Using the dedicated FEELnc program to automate the annotation of lncRNAs and their genomic classification (lincRNA, antisense, and other subclasses), the authors provided an extended set of canine lncRNAs comprising 22,880 lncRNA transcripts gathered into 10,444 gene loci. A deeper analysis of this extended RNAseq dataset revealed that, as in humans, canine lncRNAs are more tissue-specific than protein-coding genes (44 versus 17%, respectively) with 65% of all tissue-specific lncRNAs expressed in canine testis (Le Béguec et al. 2018). This catalogue was first analysed in the context of dog breed phenotypic variations, such as the "drop ear" phenotype, in which case using GWAS one lncRNA was found to be closely associated to the *MSRB3* gene involved in human deafness (Plassais et al. 2019) (see below "GWAS hits involving lncRNAs"). Furthermore, given the combined interest for lncRNAs as potential cancer drivers/biomarkers (Huarte 2015; Vancura et al. 2021) and dogs as natural and thus immunocompetent models for cancer analyses (Prouteau and André 2019), canine lncRNAs were analysed in three canine breeds (poodles, Labradors, and golden retrievers) predisposed to mucosal melanomas (MM). Using RNAseq in tumour and adjacent matched control tissues, more than 400 lncRNAs were shown to be differentially expressed between healthy and diseased animals, with 26 of these lncRNAs being reported to be conserved in humans (Hitte et al. 2019). In addition, while MM is a rare cancer in humans, the high frequency of MM in particular breeds enabled the identification of ~ 10 breed-specific lncRNAs, which were shown to be specifically differentially expressed in one breed versus the others (Hitte et al. 2019). Beside melanomas, a number of studies have established lncRNA atlases in canine cancers, such as B-cell lymphoma (DLBCL) (Cascione et al. 2019; Verma et al. 2015) or canine kidney cancer (MDCK) (Qiao et al. 2020) (Table 2A) and also linked GWAS hits to overlapping lncRNAs such as in hematopoietic cancers (Hédan et al. 2021).

**Table 2:** LncRNA studies associated with trait-related tissues in dog and livestock species

Tissues	Related traits/disease	Species	References
A. Dog			
Retina	X-linked progressive retinal atrophy	Dog	(Appelbaum et al. 2020)
Various	Breed morphology (*e.g. *"drop ear")	Dog	(Plassais et al. 2019)
Mucosal and skin tissues	Mucosal melanoma	Dog	(Hitte et al. 2019)
Lymph node	Lymphoma	Dog	(Verma et al. 2015; Cascione et al. 2019)
B. Three major species: pig, chicken, and cow*			
Muscle	Growth performance and meat quality	Pig	(J. Sun et al. 2017; Zou et al. 2017a, b; Zou et al. 2017a, b; Li et al. 2020)
		Chicken	(Li et al. 2012; Li et al. 2019; Ren et al. 2018a, b; Cai et al. 2017)
		Cow	(Choi et al. 2019; Li et al. 2020)
Mammary gland	Milk production and quality	Cow	(Tong et al. 2017; Yang et al. 2018; Ibeagha-Awemu et al. 2018; Zeng et al. 2019)
Immunity tissues	Disease or resistance against pathogenic infections	Pig	(Fang et al. 2019)
		Chicken	(Qiu et al. 2017; Hu et al. 2018; Ren et al. 2018a, b; You et al. 2019; Dai et al. 2019; Li et al. 2021; Zhang et al. 2021)
		Cow	(Özdemir and Altun 2020)
Male sexual organs	Male reproduction traits	Pig	(Esteve-Codina et al. 2011)
		Chicken	(Liu et al. 2017a, b; Zou et al. 2020)
		Cow	(Wang et al. 2019a, b; Gao et al. 2019)
Female sexual organs	Female reproduction traits	Pig	(Wang et al. 2016; Wang et al. 2019a, b)
		Chicken	(Liu et al. 2018; Adetula et al. 2018; Peng et al. 2019; Yin et al. 2020; Zou et al. 2020)
Liver and adipose tissues	Body lipid reserves and metabolic efficiency	Pig	(Wang et al. 2017; Miao et al. 2018; Kumar et al. 2019)
		Chicken	(Muret et al. 2017; Zhang, et al. 2017; Zhang 2017a, 2017b; Wu et al. 2018; Xu et al. 2019; Muret et al. 2019; Chen et al. 2019; Ning et al. 2020)
		Cow	(Nolte et al. 2019; Kong et al. 2020; Alexandre et al. 2020)
Intestine	NA	Cow	(Weikard et al. 2018; Nolte et al. 2019)
Spleen	NA	Pig	(Che et al. 2018)
		Chicken	(You et al. 2019)
C. Other livestock species*			
- Liver and cerebral parietal lobe - Placenta - Eight tissues		Horse	(Dahlgren et al. 2020; Pu et al. 2020; Scott et al. 2017)
- Skin - Endometrium - Ovary and follicle		Goat	(Ren et al. 2016; Hong et al. 2020; Lian et al. 2020; Zhao et al. 2020)
- Multiple tissues - Wool - Pituitary - Oocyte development - Consensus set of ruminant lncRNAs		Sheep	(Bakhtiarizadeh et al. 2016; Yue et al. 2015; Zheng et al. 2019; Yang et al. 2020; Wang et al. 2020; Bush et al. 2018) consensus set of ruminant lncRNAs provided by Bush et al. 2018
- Muscle - Adipose tissue - Skin - Embryos		Rabbit	(Kuang et al. 2018; Wang et al. 2018; Zhao et al. 2019; Ding et al. 2021; Kuang et al. 2020)
- Ovary - Brain, lung and spleen - Embryo fibroblast cells		Duck	(Ren et al. 2017a, 2017b; Lu et al. 2019; Y. Lin et al. 2020a,b)
- Testes - Ovary		Geese	(Ran et al. 2021; Ouyang et al. 2020)

^*^Updates from two previous reviews (Weikard et al. 2017 and Kosinska-Selbi et al. 2020)

### Long non-coding RNA studies in farm animals

Concerning livestock species, artificial selection programs, including recent genomic selection methods, have led to spectacular gains in economically important traits over the last decades (Hill 2016). However, there is little understanding of the biological mechanisms underlying such phenotypes, the knowledge of which could offer new margins of progress, such as, making genomic selection methods more robust or better exploiting the genotype-environment interactions. Therefore, a new goal of the scientific community in the animal genetic field is to provide the functional annotation of the genomes of farm animals to elucidate the hundreds of thousands of GWAS signals [160 659, 31 455, and 12 783 in the three major livestock species of cow, pig, and chicken, respectively (Hu et al. 2019)], which are known to be mainly located outside the ~ 20,000 coding regions. Chicken was the first species with a large genome to be sequenced in 2004, just after those of human and mouse (International Chicken Genome Sequencing Consortium 2004). However, the knowledge of non-coding regions in farm animals has not kept up with that in humans. Until December 2015 (Ensembl version 83), no lncRNAs were described for chicken and cow and only 135 were reported for pig, as contrasted with 14,896 and 6830 lncRNAs reported in human and mouse, respectively. This poor knowledge of the non-coding genome annotation has led to a coordinated international action to accelerate genome to phenome, termed the Functional Annotation of Animal Genomes (FAANG) project, whose aim was to produce comprehensive maps of functional elements in the genomes of livestock species to better decipher the genotype to phenotype relationships (Andersson et al. 2015). As part of FAANG, two studies have recently provided a multispecies lncRNA annotation using 8 tissues of 2 biological replicates of 3 species, namely chicken, pig, and cattle (Kern et al., 2018) and 3 tissues of 4 biological replicates of 4 species, namely chicken, pig, goat, and cow (Foissac et al. 2019).

The first lncRNAs in the three major livestock species were detected in the male gonad (Esteve-Codina et al. 2011), muscle (Li et al. 2012), and skin of the pig, chicken and cow, respectively, in the early 2010s. Since 2015, the number of publications regarding these three species has been constantly growing, with most of them focusing on the tissue-specific expression of lncRNAs or their differential expression between breeds or animal groups contrasted for an economically important trait in the species of interest (Table 2B). LncRNA studies have also been conducted in other livestock species, such as goat, sheep, rabbit, horse, as well as in other avian species, such as duck or geese (Table 2C). However, to our knowledge no studies have been performed in turkey and quail despite the identification of 1038 and 5090 lncRNAs in these two species, respectively, in the latest Ensembl annotation version (v104).

In most of these studies, a few lncRNAs have been highlighted from the lncRNA catalogues as associated to the trait of interest because of their significant differential expression between two animal groups of interest and their co-expression with a close protein-coding gene that can be used as a proxy to infer possible functions for the lncRNA, especially when the lncRNA is conserved in multiple species. For instance, the *linc-SABT1* (that should be renamed to *SATB1_DT*) has been associated with resistance to Marek’s disease (MD), because of (i) its high expression in infected birds of the Marek’s disease resistant line, and (ii) its location in the divergent orientation of the *SATB1* gene known to regulate chromatin structure and control a large number of immunity genes (He et al. 2015). The *DHCR24-DT* has been associated with lipid metabolism because of (i) its differential expression in 2 divergent lines selected for body adiposity, (ii) its location in a divergent orientation of the *DHCR24* gene coding for a key enzyme of the cholesterol synthesis in chicken and human, and (iii) its high hepatic co-expression with this mRNA gene in several chicken lines (layers and broilers) analysed at different ages (young and adult stage) (Muret et al. 2017).

### Comprehensive atlas based on multi-tissue studies in farm animals

Starting in the mid-2010s, a number of multi-tissue studies has been performed in the three main livestock species with the aim to provide more comprehensive annotation of lncRNAs given their high level of tissue specificity. *In cow,* Koufariotis et al. provided a catalogue of 9778 lncRNA transcripts resulting from the RNAseq analysis of 18 tissues, which were sampled from a single lactating cow (Koufariotis et al. 2015). *In pig,* in addition to different studies focusing on the detection of lncRNAs in various tissues, the Pig LncRNANet database (http://lnc.rnanet.org), is the most comprehensive pig lncRNA catalogue to date (Liang et al. 2018). This database contains 53,468 lncRNAs, of which 30,175 lncRNAs were retrieved from published studies and extended by 23,293 non-overlapping lncRNAs from NONCODEV4*. In chicken,* the most comprehensive lncRNA catalogue provided by Jehl et al*.*, was built using the Ensembl gene atlas as reference that was extended by non-overlapping lncRNAs from four public databases (NCBI, NONCODE, ALDB, Fr-AgEncode) and other lncRNAs modelled from a few hundred RNAseq samples using cufflinks for gene modelling and FEELnc for lncRNA prediction (Jehl et al. 2020). This extensive chicken atlas is renewed at each important update of the Ensembl annotation including significant changes in the numbers of gene loci or new genome assembly version. To date, two versions related to the two last chicken genome assemblies and Ensembl annotation *("Galgal5-Ensemblv94"* + *"GRCg6a-Ensemblv101 (equivalent to v104")* are available at http://www.fragencode.org/. The Ensembl reference has grown from around 5000 to more than 25,000 lncRNAs, of which 59% and 41% with an expression level ≥ 0.5 and ≥ 1 Transcript Per Million (TPM), respectively, in at least one of the 25 analysed tissues. Additional annotations, such as their tissue specificity, their distance and transcription orientation with respect to other closest genes, their status to a microRNA host gene (HG) are also provided for each lncRNA. For example, the chicken *MIR155HG* has been newly modelled in this atlas and found to be associated with immune functions because of (i) its hosting role for the immunity-related *MIR155*, which is conserved in human and mouse, (ii) its high expression in immune tissues (spleen, thymus, bursa of Fabricius, harderial gland) like *MIR155*, and (iii) its coexpression with immunity-related protein-coding genes.

### A limited overlap between lncRNA resources

The comparison of lncRNA catalogues from different resources has revealed a limited overlap between mono- and multi- tissue resources or between different multi-tissue resources, including the public datasets presented above. For example, Weikard et al. indicated that only 17.5% of lncRNAs detected in bovine skin in 2013 overlapped with the "18 tissues" lncRNA catalogue published in 2015 (Weikard et al. 2017). Jehl et al. also showed a low overlap of lncRNA loci between the chicken reference Ensembl, NONCODE, NCBI, ALDB and Fr-AgENCODE (INRAE) resources used for the construction of the chicken atlas, with a maximum overlap of approximately 30% between lncRNA loci from NONCODE and ALDB (Jehl et al. 2020). These limited overlap between different resources could be explained by three main explanations.

The first one is due to the low expression of lncRNAs, which are known to be globally 10- to 20-fold less expressed than mRNA transcripts both in human and domesticated animals (Derrien et al. 2012; Le Béguec et al. 2018; Jehl et al. 2020). Therefore, for each sequenced sample, the sampling of the reads has a stronger impact on the annotation of captured lncRNAs as compared to the more highly expressed mRNAs, because of the rarity of the lncRNAs in the population of sampled transcripts. The low expression level of lncRNAs is illustrated in Fig. 2A with the analysis of a RNAseq dataset of different chicken tissues mapped on the lncRNA-enriched atlas previously described (with around 20,000 mRNA loci and 25,000 lncRNA loci). Using four different tissues, it showed that the majority of reads mapped to mRNA loci with more than 80% (95.7%) of the mapped reads aligning to the 25% of the most expressed (100%) genes, all of them being protein-coding genes.

The second reason involves the higher tissue-, temporal-, and condition-specificity of lncRNA expression profiles as compared to mRNAs (Derrien et al. 2012; Le Béguec et al. 2018; Jehl et al. 2020), making critical the number and origin of samples to be analyzed. So far, the reference annotations provided by Ensembl or NCBI/RefSeq for domesticated species are based on a very few set of RNAseq samples (e.g. for chicken, 21 samples from a unique project for Ensembl and 129 samples from different projects for NCBI/RefSeq) in comparison to the thousands of RNAseq samples generated over the past decade and publicly available in ENA or SRA databases. Therefore, these reference gene sets do not recapitulate the diversity of tissues, ages and physiological stages of lncRNA expression patterns. Consequently, lncRNA gene models are highly sample-dependent in comparison to more broadly expressed mRNAs, as illustrated by the little overlap of lncRNA loci between Ensembl and NCBI/RefSeq (about 13.7%), whereas almost all mRNA loci are common to both resources (87%) (Fig. 2B).

Finally, as previously illustrated in Table 1, lncRNA databases also make use of different bioinformatic tools at each step of the lncRNA annotation process (Table 1). This most likely influences gene structure boundaries (especially given the limitations of tools for the reconstruction of full transcripts from short-read RNAseq) together with the correct attribution of gene biotypes (mRNA versus lncRNA), and therefore, the extent of overlap between lncRNA sets.

In conclusion of this section, unlike protein-coding genes, genome annotation for lncRNAs (transcript and gene loci) requires considering the entire diversity of tissues, stages, conditions available in public sequences databases. In combination with standard computational procedures and benchmarked tools, the inclusion of many more projects and associated RNAseq samples within the same species both using short-read RNASeq and, in the coming years, long-read RNAseq technologies will most likely increase the completeness of lncRNA sets in domesticated animals.

## Long non-coding RNAs and comparative genomics

Comparative genomics, defined as the comparative study of the structure and function of the genomes of different species, is a common method to identify new genes and their functions, and thus to more accurately annotate new genomes (König et al. 2018). However, although the approaches used for protein-coding genes are quite efficient, they have been revisited for the long non-coding genes (lncRNAs) due to their structural and functional specificities.

Over the past decade and linked to the growing interest for lncRNAs, multiple studies have used comparative genomic approaches to detect and annotate novel lncRNAs across phylogenetically divergent species. (Necsulea et al. 2014; Hezroni et al. 2015; Sarropoulos et al. 2019). However, a set of annotated genomes and a bioinformatic method to compute the distance/similarities between the source and target genomes are required. So, even though the catalogues of lncRNAs in many species have been increasing, especially due to the standardization of RNAseq-based methods, lncRNA repertoires of domesticated species remain mostly incomplete, as underlined before. If the incomplete annotation of lncRNAs represents one of the issues for the comparative study of conserved lncRNAs, the phylogenetic divergence between targeted species is also an important parameter to be considered.

Indeed, lncRNAs evolve very fast and, usually, the higher the evolutionary distance between two species, the fewer the number of orthologous lncRNAs (Bu et al. 2015; Chen et al. 2016; Hezroni et al. 2015; Kern et al. 2018; Washietl et al. 2014; Necsulea et al. 2014). Moreover, the rates of birth and death of lncRNAs seem to be very high, even in closely related species, as shown by Kutter et al. in rat and mouse species, where half of the intergenic lncRNA loci have been gained or lost since the last common ancestor (20 My) (Kutter et al. 2012). And so some lncRNAs might appear as derived from a lost protein-coding gene (Duret 2006; Hezroni et al. 2017). Finally, even if the genomic sequence of a lncRNA is conserved, its expression profile in matched tissues might differ between species (comparative transcriptomics) (Washietl et al. 2014).

**Fig. 3:**
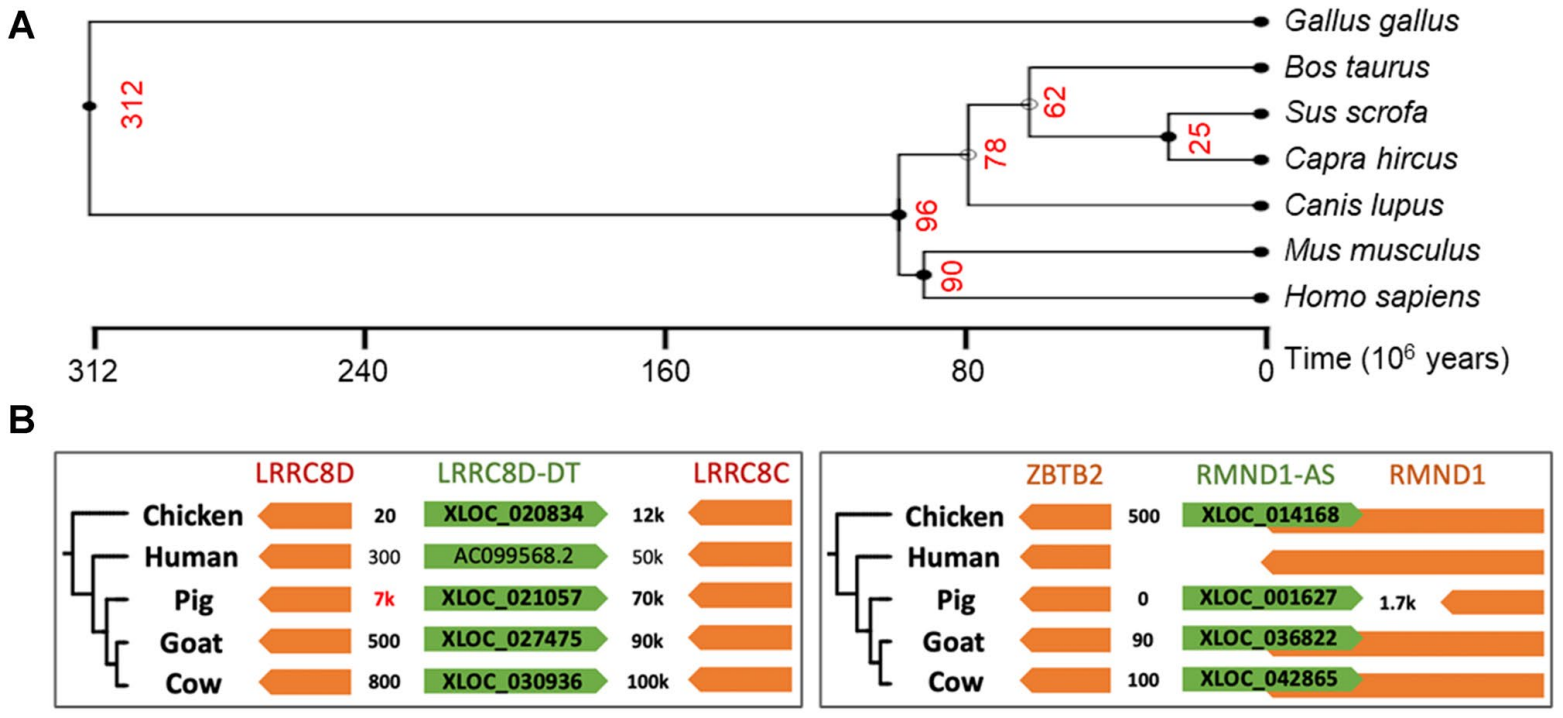
Phylogenetic divergence between domesticated species, mouse, and human. **A** Red numbers correspond to the common ancestor of different species. This tree was generated using the TimeTree database (Kumar et al. 2017). Distances were calculated from estimated molecular time. **B.** Genomic conservation of 2 lncRNAs (in green) in divergent position extracted from Foissac et al. (Foissac et al. 2019)

In the case of the domesticated species, these evolutionary distances are quite heterogeneous (Fig. 3A). Indeed, even though most of the species of the "domesticated" group diverged from human ~ 96 mya, the evolutionary distances within the group are very variable. For example, the closest species are "goat" and "cow" that share a common ancestor around 25 mya., whereas "pig" diverged 62 mya. The chicken appears as an outlier because it diverged 300 mya. Interestingly, some lncRNAs appear to be conserved over a large time-scale possibly due to their common function in all eukaryotes (Kern et al. 2018; Wiberg et al. 2015).

Based on all these observations and considering the availability of adequately annotated genomes, several-related approaches have been used to perform comparative genomic analyses of lncRNAs. The first one, which was usually used for protein-coding genes, is based on the alignment of the primary sequences of genes on the target genome. However, although this technique works relatively well for mRNAs, it needs to be adapted for lncRNAs. Overall, around 70% of lncRNAs have no sequence orthologues (e.g. given a certain threshold of sequence similarity and alignment length) in species that have diverged for over 50 mya (Hezroni et al. 2015). Furthermore, not all parts of a lncRNA sequence evolve at the same rate. LncRNA exons are more stable than intergenic sequences and mRNA introns (Cabili et al. 2011). So, only a few "patches" of sequences (*e.g.* short conservation islands), potentially corresponding to RNA or protein binding regions, seem to be conserved and are generally located in lncRNA exons and promoters (Noviello et al. 2018; Darbellay and Necsulea 2020). These patches are significantly shorter than those located in mRNAs, are found in only one or two exons, and can tolerate large rearrangements. Quinn et al. considered that only 10% of the sequence might be sufficient to support the function of a lncRNA (Quinn and Chang 2016). Recently, a new tool, called lncLOOM, based on a graph representation of a multiple sequence alignment (MSA) and integer linear programming, has been published for the functional prediction of lncRNA short motifs positionally conserved between species (Ross et al. 2021). Applied to vertebrate species, the tool allowed the identification of functional domains in known lncRNAs, such as *Cyrano* and *CHASERR,* as well as in the 3′-UTR of protein-coding transcripts (Ross et al. 2021).

However, while lncRNA gene structures change rapidly and might therefore be an obstacle to the detection of homologous sequences, other important features can be used in the detection of lncRNAs by comparative genomics. Indeed, lncRNAs are more tissue specific than protein-coding genes, which can help refine predicted functions (Guttman et al. 2011). Such a characteristic shows the importance of working with matched tissue(s) between species in the case of comparative transcriptomic approaches. Interestingly, the oldest conserved lncRNAs are generally expressed in tissues related to embryonic development (Necsulea et al. 2014; Washietl et al. 2014). Another major attribute of the biology of lncRNAs is related to their positional conservation (synteny) between species genomes. This trend has been observed between human and mouse, as well as in the case of comparative genomic analysis of domesticated animals (Foissac et al. 2019) (Fig. 3B). A possible explanation could be their potential function related to gene regulation through the reorganization of local chromatin structure. To identify such positionally conserved lncRNAs, the identification of positionally conserved neighbour genes, usually mRNAs, is initially required; if these genes are orthologous in the targeted species, they will also define a conserved syntenic interval for lncRNAs. Using this strategy, a few studies have found positionally conserved lncRNAs within distant species (Hezroni et al. 2017, 2015; Sarropoulos et al 2019; Muret et al. 2017, 2019; Jehl et al. 2020).

**Fig. 4:**
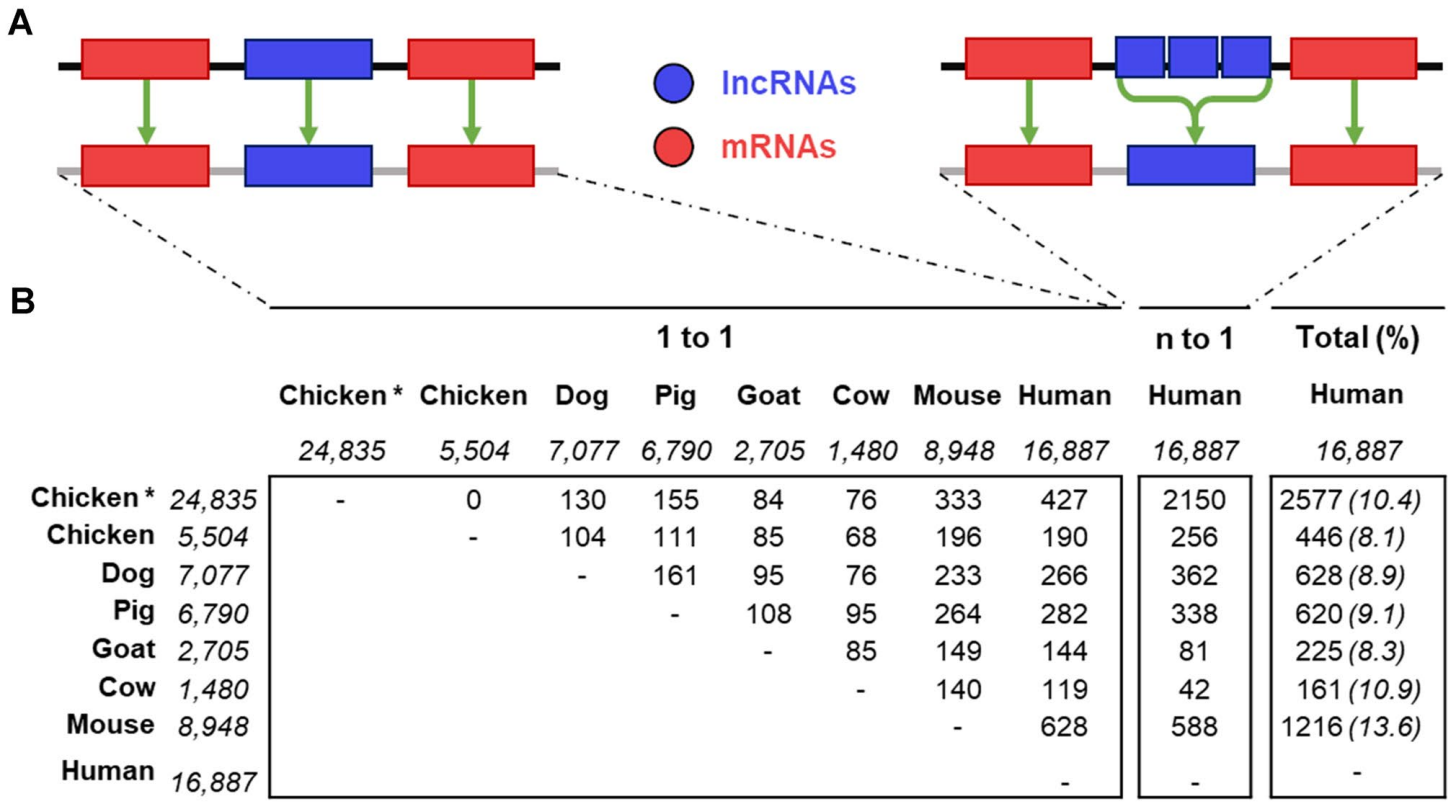
Syntenic conservation of lncRNAs across 7 species. **A** schema of "1–1" and "n–1" principles of positionally conserved lncRNAs. The "1–1" corresponds to the case of a strict and unique syntenic equivalent in both species located in-between two adjacent "1–1" protein-coding genes. The "n–1" corresponds to the case of multiple lncRNA loci in the analysed species that corresponds to an unique lncRNA in human located between the two "1–1" protein-coding genes. **B** Number of lncRNA for each homology category across species with numbers of lncRNA loci (in italic) extracted from Ensembl (v104). The "*" indicates the chicken lncRNA-enriched annotation anchored on the v101 (equivalent to v104) Ensembl resource (Jehl et al. 2020)

Using a similar approach, we estimated the number of syntenic lncRNAs among seven species including domesticated species (except horse), mouse and human (Fig. 4B). As depicted in Fig. 4A, we have searched for lncRNAs corresponding to strict one-to-one equivalences (termed "1–1") for all the species-pairs. In a second step, we considered the ''n–one'' orthologous lncRNAs ("n–1") defined as n adjacent lncRNA loci in one of the six species related to a single syntenic lncRNA in the human species which is considered here to be the species with the most accurate annotation of lncRNAs.

As expected, the smaller the phylogenetic distance between species, the higher the number of orthologous lncRNAs. For instance, we observed with the human species between 190 and 628 orthologous ''1–1'' lncRNAs for the chicken and mouse species, respectively. For the other livestock species, between 119 Ensembl lncRNAs in cow and 282 lncRNAs in pig can be considered as syntenically conserved with a human lncRNA using the strict definition ''1–1''. It is important to note that the comprehensiveness of a species-specific lncRNA catalogue has a major impact on the number of annotated orthologous lncRNAs. Indeed, we observed 427 (2150) versus 190 (256) ''1–1'' (''n–1'') orthologous lncRNAs between chicken and human species when comparing the two lncRNAs chicken atlas (v104 Ensembl catalogue versus lncRNA-enriched v104 Ensembl catalogue from Jehl et al. 2020). The increase in the number of ''n–1'' versus ''1–1'' orthologous lncRNAs for chicken, pig, and dog species is probably due to less accurate modeling of gene structures in these species compared to that in humans where transcript boundaries are validated by manual curation and 5′/3′ experimental supports (*e.g.* CAGE and polyA signals); the n gene would correspond to only one gene or some of them would actually 5′ or 3′ UTRs of neighboring protein-coding genes (Muret et al. 2019). Interestingly, the sum of the ''1 to 1'' and ''n to 1'' orthologous lncRNAs between each domesticated and human species is around 10% of the total lncRNAs in each species (Fig. 4B, right column) as reported in individuals studies of diverse species (Le Béguec et al. 2018; Kevin Muret et al. 2019; Breschi et al. 2017).

In conclusion, compared with the direct annotation of lncRNA gene structures, comparative genomic approaches allow strengthening the annotation of lncRNAs by providing insights into potentially functional lncRNAs related to a shared trait/disease, even though phylogenetic divergences should be considered for measuring the conservation of lncRNAs.

## Long non-coding RNAs and transposons: towards long-read sequencing?

One of the most intriguing aspects of lncRNA biology lies in the observation that their sequences are highly enriched in transposable elements (TEs), that is, repetitive mobile elements capable of copying and moving into genomes. Briefly, TEs can be classified into two classes based on the mechanism by which they integrate into genomes. The first class, defined as retrotransposable elements, make use of a "copy-and-paste" strategy via the production of an intermediate RNA molecule, which is reverse transcribed into cDNA in order to be inserted into the genome. Usually, class 1 is subdivided into long terminal repeat (LTR) and non-LTR according to the biochemical mechanism of chromosomal integration, with non-LTR regrouping short and long interspersed nuclear elements (SINEs and LINEs). The second class of TEs, corresponding to DNA transposons, are mobilised into genomes through a "cut-and-paste" strategy whereby a DNA intermediate is produced. In humans, more than 80% of lncRNAs overlap at least one annotated TE, with 40% of lncRNA sequences being derived from TEs (Kelley and Rinn 2012; Kapusta and Feschotte 2014). This led some authors to hypothesise that TEs are the functional domains of lncRNAs (Johnson and Guigo 2014). Indeed, it has recently been shown that specific repeat families can drive nuclear retention of lncRNAs in humans (Lubelsky and Ulitsky 2018; Carlevaro-Fita et al. 2019) or regulate mRNA translation (Zucchelli et al. 2015).

**Fig. 5:**
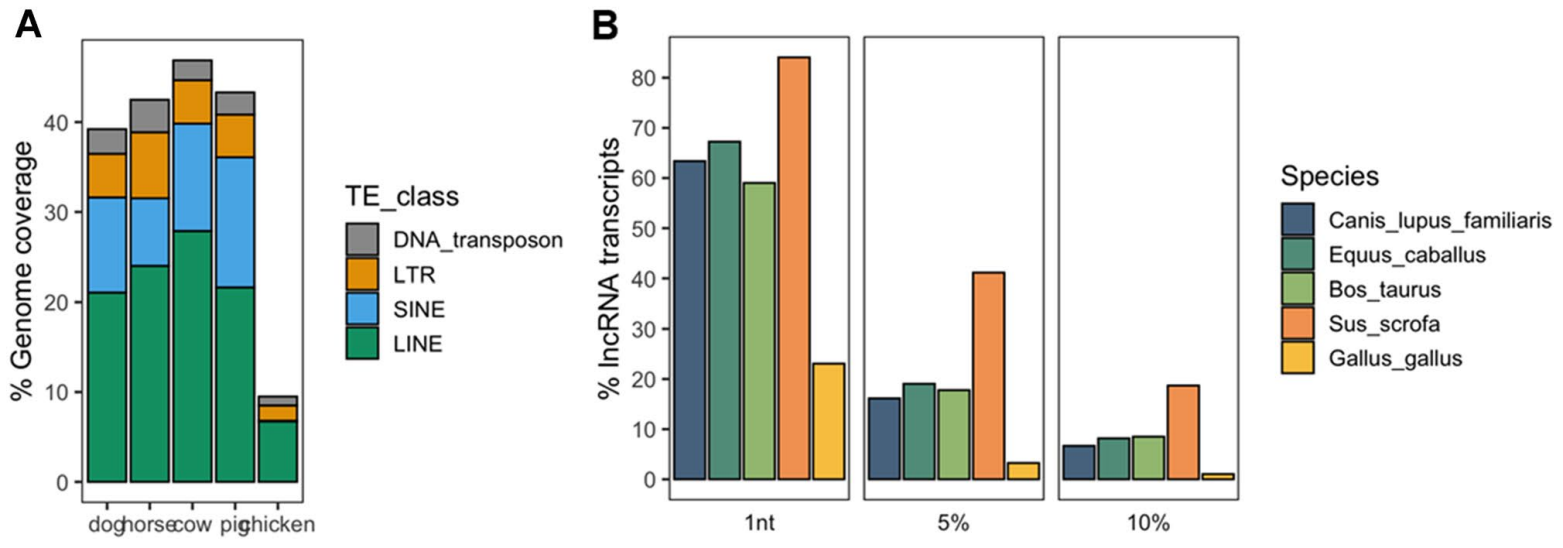
Association between transposable elements (TEs) annotated by RepeatMasker and long non-coding RNAs annotated by Ensembl (v103) in 5 genome assemblies (canFam3, equCab3, bosTau9, susScr11 and galGal6). **A** Proportion of the genome covered by four TEs classes: LINEs, SINEs, LTRs, and DNA_transposons in green, blue, orange, and grey, respectively. **B** Proportion of Ensembl-based lncRNA transcripts overlapped by TEs for three fractions overlap (≥ 1 nucleotide, ≥ 5%, and ≥ 10% of the lncRNA sequence) in five domesticated species (dog, horse, cow, pig, and chicken, respectively, in dark green, orange, purple, pink, and light green)

Regarding the 5 domesticated species studied in this review, the proportion of each reference assembly covered by TEs annotated by the RepeatMasker (http://www.repeatmasker.org) varies from 9.5% for chicken (galGal6) to 46.8% for the cow (bosTau9) (Fig. 5A). The lower proportion of TEs in the chicken genome could possibly be explained by the low copy numbers of SINE elements (< 10,000) compared with other mammals, such as humans (> 1,500,000) (Kapusta and Suh 2017). More specifically, SINE retrotransposons cover less than 0.1% of the chicken genome (7.6 Mb) as compared, for instance, to 10.5% (253 Mb) and 14.4% (359 Mb) for dog and pig genomes, respectively. When intersecting the annotations of lncRNAs and mobile genetic elements, between 23% of lncRNA transcripts for chicken and 84% for pigs are overlapped by at least one TE (Fig. 5B). In addition, when increasing the fraction of lncRNA transcript sequences that are overlapped by TEs, pig lncRNAs are still remarkably different from those of other mammals, with 41.1% and 18.7% of pig lncRNA sequences being composed of at least 5% and 10% of transposable elements, respectively (Fig. 5B). The inclusion of long-read transcriptomic data in the Ensembl-based annotation of pig lncRNAs has probably allowed a better reconstruction of lncRNA transcripts embedding repetitive elements such as TEs (See Fig. 5).

In line with this observation, recent transcriptome sequencing studies using long-read RNAseq (LR-RNAseq) promise to revolutionise annotation methods. Indeed, all reads from short-read RNAseq (SR-RNAseq) that are shorter than a specific repeat length will, by definition, not be uniquely assigned to one genome position, and thus would be considered as "multimapped". This can have a major impact on transcriptome reconstruction, especially for repeat-associated transcripts, such as lncRNAs. Steijger et al. showed that the best-performing method for reconstructing transcript models based on SR-RNAseq identified at most 21% of spliced transcripts in humans (Steijger et al. 2013). More recent studies involving the capture of lncRNAs followed by LR-RNAseq highlighted novel features for human and mouse lncRNA gene structures with (i) extensions of their 5′ and 3′ ends, (ii) similar splice length and exon count as in mRNAs (Lagarde et al. 2017), and (iii) near universal splicing of non-coding exons (Deveson et al. 2018). In addition to transcript structure, LR-RNAseq can allow the improved quantification of repeat-associated transcripts compared with SR-RNAseq (Sessegolo et al. 2019; Workman et al. 2019). Given that LR-RNA sequencing technologies represent an unfragmented vision of the transcriptome, they will more likely also facilitate gene reconstruction in domesticated species by direct exon/exon connectivity and read spanning repeats.

## Functions of long non-coding RNAs in domesticated species

As we have seen, lncRNA annotations have been associated with contrasted conditions, genotypes or GWAS hits (Table 2). However, as for human or model species (Bassett et al. 2014), assigning a functional mechanism to a lncRNA also remains a difficult task in domesticated species. Regarding GWAS, the first issue lies in identifying the causative variant in the GWAS region: in general, several polymorphisms being in linkage disequilibrium in the GWAS interval without the possibility to target the causative one(s) because of the low number of contrasted phenotypes which are observed (*i.e.* meiosis). When the mutation is located outside of a gene body, the second obstacle is to determine which gene is regulated by this polymorphism since the regulatory elements (*e.g.* enhancers) can act distantly from the targeted gene. Finally, the last difficulty is to validate the impact of the lncRNA gene (containing the polymorphism or regulated by this one) on the phenotype of interest. This last difficulty can be generalized to different observational levels such as animal, tissue, cell phenotypes. Thus, although tens of thousands of lncRNAs have been identified in the genomes of animals, their functions remain mostly unknown, irrespective of species. A review in 2019 reported that only 60 lncRNAs were involved in lipid metabolism despite the high number of lncRNAs identified in related tissues (e.g. liver or adipose tissue); these lncRNAs were mainly described in human or mouse, with only a precise described mode of action for a few of them (Muret et al. 2019). The main reasons probably stem from (i) an incomplete characterization of lncRNA isoforms and promoter sequences, (ii) a poor knowledge of the functionally important patches of lncRNA sequences, (iii) a lower expression level, and finally iv) multiple modes of action with cis or trans effect (Bassett et al. 2014).

In summary and as illustrated in the next paragraphs, only the function of a little number of lncRNAs has been elucidated in domesticated species.

### GWAS hits involving lncRNAs

So far, there are only a few studies that have pinpointed lncRNAs located in GWAS intervals associated with a particular disease or trait. These studies have combined different approaches based on either genetic interval refinement using additional animals (and therefore meiosis) and/or molecular experiments to more deeply conclude the causative status of the lncRNA, although never with a formal demonstration with an in vivo experiment.

Concerning the dog species, Plassais et al. identified a ~ 1.5 Mb locus after GWAS involving hunting dog breeds affected by Human Sensory Autonomic Neuropathy (HSAN). After targeted DNA sequencing of the locus in four breeds, one exonic point mutation in an intergenic lncRNA termed *GDNF-AS* (transcribed in antisense orientation of the *GDNF* gene) was identified in affected dogs and absent in a panel of > 800 healthy dogs. By qRT-PCR analysis, a significant decrease of both the lincRNA and the mRNA expression levels was observed in specific tissues (*e.g.* dorsal root ganglia). In addition, gel shift assay (EMSA) revealed that the mutation significantly altered the binding of a transcription factor, altogether suggesting that *GNDF-AS* functions as an enhancer RNA (eRNA).

Concerning the livestock species, we can cite the callipyge (CLPG) locus responsible for muscle hypertrophy in sheep in which the *CLPG* mutation has been deeply studied and shown as interacting in trans between a maternally expressed repressor lncRNA, *MEG3* (alias *GTL2),* and its paternally expressed hypertrophy-promoting target, *DLK1* (Georges et al. 2003).

Another example concerns the Celtic Polled locus in cattle. Initially, a rather limited candidate region of 400 kb was identified by GWAS but contained numerous candidate polymorphisms. The study of new cases with versus without phenotype combined with different genotyping strategies allowed to reduce the number of candidate polymorphisms to a single one, the causal mutation (PC/c). The qRT-PCR analysis of the 7 genes located in the 500 kb upstream and downstream of the PC/c mutation revealed only one gene a differentially expressed between PC/p polled versus WT animals, a lincRNA without known function (Allais-Bonnet et al. 2013).

Other lncRNAs have been associated with a trait of interest by GWAS but these association studies require further investigations to confirm their phenotypic causality status because of the many SNPs in linkage disequilibrium. For instance, we can mention the lncRNAs *pouBW1* (Mei et al. 2016) or *pouMU1* (Ren et al. 2017a, b) related to chicken growth or the *lncRNA8138.1* related to reproductive traits.

### Functional analysis by molecular biology approaches

#### Functional analysis by knock-out and knock-down

##### Validation of a single long non-coding RNA candidate

Pioneer researchers studying specific lncRNAs have recycled methods initially developed for other classes of RNAs, such as tRNAs and mRNAs. To assign functions to lncRNAs, geneticists have successfully generated knock-outs (KO) or knock-downs (KD) of lncRNAs in cells or animal models (Knott and Doudna 2018). However, these targeting approaches have given rise to two main considerations regarding lncRNA specificities. Generating a lncRNA KO by deleting any exon without knowing its functional status could be risky. A more radical approach would be to delete the whole lncRNA gene or target the lncRNA promoters. In the last case, it is important to (i) verify that this promoter is not shared with another gene as in the case of bidirectional lncRNAs (Zhu et al. 2016), (ii) to evaluate the expression levels of neighbouring genes, and (iii) to perform rescue experiments.

A lncRNA depletion could be achieved using sequence-specific antisense oligonucleotides (ASO) able to target nuclear lncRNAs in contrast to small interfering RNAs (siRNA), thus efficiently knocking them down through the promotion of their RNAse H degradation (gapmers) (Crooke et al. 2021). The main pitfall relies on the efficient targeting of the lncRNA isoform of interest by short ASO (16–24 nucleotides) and could require preliminary experiments to determine the different transcript isoforms of the studied model.

##### Screening approaches

To more systematically identify the functional role of lncRNAs, a screening approach might be sometimes attempted in parallel to high-throughput RNA-sequencing. CRISPR libraries for all human protein-coding genes (~ 20,000 genes) are available from non-profit companies (*e.g.* Addgene) for the performance of loss of function (CRISPR KO), gain-of-function (CRISPR activator, CRISPRa), or mRNA knockdown studies via CRISPR inhibition (CRISPRi) at a modest cost (< 500 €). These libraries, containing 3–10 single guide RNAs (sgRNAs) per targeted transcript, have been validated in various studies (Konermann et al. 2015; Joung et al. 2017). However, CRISPR KO libraries seem inappropriate for lncRNAs, as the functional domain(s) of lncRNAs have not been yet clearly identified. In contrast, CRISPRa and CRISPRi strategies (Liu et al. 2017a, b; Esposito et al. 2019) could efficiently modulate the expression (up- or downregulate) of lncRNAs; however, 2 main limitations need to be mentioned. First, the single guide RNA (sgRNA) libraries have been designed from lncRNA databases, such as Ensembl or GENCODE, built on models reconstructed from RNAseq data of different cell types or differentiation states and therefore not specific for a given cell type/tissue; thus, many sgRNA might not be functional in the studied cell model given the high tissue- and condition-specific feature of lncRNAs. Second, the design of a sgRNA library might be sometimes hazardous because of the imperfect knowledge of lncRNA promoter regions, despite the recent advancements in 5′ end annotation in human, dog, and chicken (Hon et al. 2017). To the best of our knowledge, such CRISPR libraries are not yet available for domesticated species.

Even if these two strategies (KO & KD) are correctly evaluated, other complementary experiments would still be required to establish the mode of action of these lncRNAs.

#### Long non-coding RNA interacting partners

The functions of lncRNAs have been previously reviewed (Quinn and Chang 2016; Gil and Ulitsky 2019; Statello et al. 2021). Their functional mechanisms are diverse, including lncRNAs that act as scaffolds, decoys, or signals. In addition, they can act by regulating in both cis or trans (Ulitsky and Bartel 2013; Geisler and Coller 2013).

##### Interacting partner detection

Numerous methods have been developed to identify the interactions of lncRNAs with either RNA, DNA, or proteins (Goff and Rinn 2015). Despite their differences, the principle is often the same requiring, the enrichment of lncRNA partners using lncRNA precipitation. Most groups performed lncRNA precipitation using short oligonucleotides coupled to biotin. Based on complementary base-pairing, ribonucleotide complex-associated to the biotinylated ASO were purified via streptavidin beads followed by stringent washes. The identity of the partner was revealed using sequencing analyses (RNA or DNA) or spectrometry (proteins). As with all enrichment experiments, false positives and false negatives are inherent to these approaches, rendering the performance of validation experiments a crucial step. When an lncRNA-interactant is identified, complementary experiments are needed to validate the domain of lncRNA interacting with a protein or an RNA or a DNA sequence. Depending on the lncRNA-interactant nature, different experiments can be envisaged.

##### Interaction domain identification

While robust, the conventional protein immunoprecipitation followed by lncRNA detection (RT-qPCR) requires an efficient crosslinking between the lncRNA and the protein (before IP), which is not always possible in animal models. A biotinylated short-RNA complementary to the RNA interactant is usually used as a bait for the successful purification and detection of lncRNA-RNA interactions using streptavidin beads. Similar approaches are used for DNA, but involve an efficient DNA fragmentation or partial digestion using recombinant restriction enzymes (Chu et al. 2015).

##### Validation of the interacting domain by inhibiting interaction

An elegant detection strategy works by preventing the binding between the candidate partner and the studied lncRNA. This can be achieved by protecting or deleting the interacting domain of the lncRNA. The second strategy is based on the prime-editing approach published in 2019 (Anzalone et al. 2019). This CRISPR 3.0 method allows researchers to rewrite the DNA sequence encoding the lncRNA or the putative partner. To date, this method is probably the most appropriate for studying lncRNA domains and functions because the experiments are based on the normal expression level of the lncRNA. More specifically, experiments do not require the overexpression of the lncRNA or its putative partner. Although this approach is clever, designing an efficient prime-editing sgRNA (pegRNA) is difficult (Lin et al. 2020a, b; Marzec and Hensel 2020). Given that the efficiency of a pegRNA varies between 0.1% and 50%, many clones must be sequenced before the identification of the correct edited clone (*i.e.* homozygous edition).

#### Examples in domesticated animals

As described above, RNA interaction experiments as knock-out and knock-down using CRISPR tools coupled to ASOs are well suited to elucidate the functions of lncRNAs both in vitro and in vivo. Concerning the in vitro studies (*i.e.* using a cellular system), while overexpression and knock-down experiments are reported in domesticated species for protein-coding genes, this type of studies is less frequent for lncRNAs. Table 3 provides a few studies associated with in vitro functional analyses of lncRNA for livestock species. We can note that some studies start to use ASO sequences which are more efficient to deplete the target lncRNA than siRNA. Concerning the in vivo studies allowing to formally validate the impact of a gene mutation on a phenotype, they are still limited for protein-coding genes. We can cite the disruption of the *CD163* gene in pigs by CRISPR conferring resistance to PRRSV infection, the activation of the *MSTN* gene (myostatin) in sheep and cow resulting in meat production improvement (for review, see (Menchaca et al. 2020) or the correction of muscular dystrophies in dogs using CRISPR targeting the *DMD* gene (dystrophin) (Amoasii et al. 2018). To the best of our knowledge, such studies do not yet exist for lncRNAs.

**Table 3:** LncRNA studies associated with in vitro functional analyses for livestock species

lncRNA name	lncRNA impact	Cellular model	Strategy	Year (Refs)
A. Chicken				
*MHM*	Embryonic development Sex determination	Egg (0-day blastoderms)	OverEx	2012 (Roeszler et al. 2012)
B. Cow				
*ADNCR*	Impact on SIRT1 by competing with miR-204 as a ceRNA to regulate adipogenesis	HEK293T,HEK293A & ADSC cells	OverEx KD by siRNA	2016 (Li et al. 2016)
*LncRNA candidate 1*	Embryonic developmental rates	Cattle matured oocytes	KD by siRNA	2015 (Caballero et al. 2014)
*H19*	Differentiation of satellite cells. Blocking of the Sirt1/FoxO1 pathway during myogenesis	C_2_C_12_ cells & satellite cells (from adult cattle muscle)	OverEx KD by pLenti-NTC interference vector	2017 (Xu et al. 2017)
*lnc403*	Inhibit myogenic differentiation of bovine skeletal muscle satellite cells Negatively regulated gene Myf6 and positively regulated protein KRAS	Satellite cells (from foetal bovine muscle)	OverEx KD by siRNA	2020 (Zhang et al. 2020)
*IGF2 AS*	Promote proliferation and differentiation of bovine myoblasts through various pathways	Myoblasts (from foetal bovine muscle)	OverEx KD by siRNA	2020 (Song et al. 2020)
C. Pig				
*lncIMF4*	Associated with adipogenesis and effect in intramuscular preadipocyte proliferation and differentiation	Intramuscular preadipocytes (from 2 pig breeds)	KD by siRNA	2020 (Sun et al. 2020)
*TCONS_00815878*	Decreasing of Myod, MyoG and MyHC such as glycolysis and pyruvate metabolism which are related to skeletal muscle satellite cell differentiation	Skeletal muscle satellite cells	KD by ASO	2019 (Huang et al. 2019)
*XLOC-2222497*	Regulate AKR1C1 and progesterone metabolism	Endometrial cells	OverEx KD by ASO	2020 (Su et al. 2020)

*KD* knock-down, *OverEx* overexpression

## Conclusion/perspectives

Domestic animals have been selectively bred by humans during thousands of years for cultural or economic reasons. Consequently, they provide an almost infinite space of desired phenotypes involving genomic variations in protein-coding and non-coding elements. Although the former has been studied for a long time, the importance of long non-coding RNAs has only been investigated recently in human and model organisms, and even more recently in domesticated animals. Despite the democratization of short-read RNAseq combined with efficient bioinformatic programs to manage these data, we showed that lncRNA annotations in domesticated animals are far from complete as compared to human or mouse, both in terms of number of gene loci and alternative isoforms. Moreover, the catalogues of lncRNAs available in public resources display a very low overlap. As we have seen, this can mainly be explained by the specific features of lncRNAs (high tissue-specificity, low expression levels, high repeat content, …) and the limited number of RNAseq samples used for generating these catalogues, even for dedicated annotation resources such as Ensembl or NCBI/RefSeq. Furthermore, the diverse computational solutions used by these resources probably impact the number of shared lncRNAs, by defining dissimilar gene boundaries (at the transcriptome reconstruction step) or by misclassifying transcript biotypes (at the coding-potential assessment step).

In order to leverage the importance of lncRNAs in animal models and evaluate their functionality, several complementary directions could be envisaged to increase the completeness of the annotations and to provide more accurate catalogues of lncRNAs. The first one relies on exploiting and combining the wealth of public RNASeq data available in public repositories (SRA/ENA) in order to include as many as possible tissues, physiological/pathological stages and environmental conditions. Although feasible in theory, this requires efficient programs and large computational infrastructures to regularly cope with the thousands of data now available for domesticated species and to carefully version each newly produced catalogues (Seal et al. 2020).

As mentioned previously (Steijger et al. 2013), one of the major bottlenecks in the bioinformatic process of annotating gene models can be related to the transcript reconstruction step *i.e.* the process of connecting multiple exons into correct spliced isoforms. The growing interest in long-read RNA sequencing, provided by technologies such as ONT or PacBio, will likely facilitate the reconstruction of full-length non-coding (and coding) gene models for domesticated species in the near future. Yet, these technologies still produce shallow sequencing depths compared to short-read RNAseq. This could be an issue for lowly expressed transcripts such as lncRNAs although capture strategies followed by LR-RNAseq have been recently applied with success in human and mouse (Lagarde et al. 2017).

The availability of these catalogues of lncRNAs in domesticated species, even if not perfect, has allowed researchers to include these new types of regulatory genes in their studies, by showing some of these lncRNAs to be differentially expressed across treatments, conditions, or genotypes. To go further on some lncRNAs of interest, it is important to keep in mind that multiple evidence should be considered to assess lncRNA functionality in domesticated animals. The identification of an orthologous lncRNA, by sequence or positional conservation, in human databases is a good proxy for its real existence but would involve that the phenotype of interest is evolutionary conserved between the studied domesticated species and human. While information has been gained about the evolution of lncRNAs across distantly related species through large-scale comparative transcriptomic studies, very little is known regarding the conservation of lncRNAs at smaller time-scale (e.g. between populations within a species). The genetic architecture of domesticated species, with homogeneous breed/population structure and potential large-scale phenotypic data, represent ideal models for dissecting the impact of the non-coding genome on a breed-associated trait. The combination of exhaustive/accurate lncRNA genomic maps with standardized functional technologies (e.g. ASO or CRISPR) represent a prerequisite to assess lncRNA functionality and will pave the way to decipher the role of these enigmatic transcripts in the phenotypes of domesticated animals.
